# Cross-Layer Security for 5G/6G Network Slices: An SDN, NFV, and AI-Based Hybrid Framework

**DOI:** 10.3390/s25113335

**Published:** 2025-05-26

**Authors:** Zeina Allaw, Ola Zein, Abdel-Mehsen Ahmad

**Affiliations:** School of Engineering, Lebanese International University, Al Khiyara, West Bekaa 1803, Lebanon; abdelmehsen.ahmad@liu.edu.lb

**Keywords:** 5G/6G networks, network slicing, software-defined networking (SDN), network function virtualization (NFV), AI/ML, cross-layer security

## Abstract

Within the dynamic landscape of fifth-generation (5G) and emerging sixth-generation (6G) wireless networks, the adoption of network slicing has revolutionized telecommunications by enabling flexible and efficient resource allocation. However, this advancement introduces new security challenges, as traditional protection mechanisms struggle to address the dynamic and complex nature of sliced network environments. This study proposes a Hybrid Security Framework Using Cross-Layer Integration, combining Software-Defined Networking (SDN), Network Function Virtualization (NFV), and AI-driven anomaly detection to strengthen network defenses. By integrating security mechanisms across multiple layers, the framework effectively mitigates threats, ensuring the integrity and confidentiality of network slices. An implementation was developed, focusing on the AI-based detection process using a representative 5G security dataset. The results demonstrate promising detection accuracy and real-time response capabilities. While full SDN/NFV integration remains under development, these findings lay the groundwork for scalable, intelligent security architectures tailored to the evolving needs of next-generation networks.

## 1. Introduction

Fifth-generation (5G) networks represent the latest advancements in mobile communication technologies as defined by the International Telecommunication Union (ITU). The inaugural commercial deployment occurred in Finland in June 2018, marking a transformative leap toward ubiquitous connectivity and enabling a new era of low-latency, high-speed communications. With peak data rates exceeding 10 Gbps and ultra-reliable low-latency communications (URLLC), 5G facilitates a broad spectrum of applications, from autonomous driving and smart manufacturing to remote robotic surgeries and augmented reality (AR) experiences [1]. This technological evolution is paving the way for 6G, which is expected to further integrate AI-driven automation, terahertz (THz) communication, and intelligent network orchestration to support even more advanced services [2]. However, as 5G adoption grows and 6G research accelerates, securing these networks remains a critical challenge.

The adoption of technologies like Network Function Virtualization (NFV) and Software-Defined Networking (SDN) has introduced new vulnerabilities in 5G/6G infrastructures. SDN’s decoupling of the control and data planes presents security risks, while NFV’s dynamic resource allocation expands the attack surface [3]. Moreover, network slicing—a key feature of 5G/6G—allows for customized, isolated virtual networks but raises concerns regarding inter-slice communication vulnerabilities and cross-slice attacks [4]. As network architectures grow in complexity, the number of potential attack vectors increases, necessitating advanced security mechanisms. Artificial Intelligence (AI) and Machine Learning (ML) have been widely adopted to detect, mitigate, and respond to threats in real time [5]. However, ensuring the integrity and reliability of network applications throughout their lifecycle remains a critical challenge, especially given the widespread adoption of 5G/6G across various sectors.

### 1.1. Rationale for the Study

Traditional security frameworks face significant challenges in providing holistic protection across the multi-layered architecture of 5G/6G networks. The complexity of these advanced networks, characterized by virtualization, distributed computing, and software-defined infrastructures, demands a security approach that goes beyond traditional boundaries. Existing solutions often focus narrowly on isolated threats, leaving vulnerabilities exposed across other critical layers. This fragmented approach fails to address the interconnected and dynamic nature of modern networks, where threats can propagate swiftly across layers. Furthermore, conventional security methods lack the flexibility to counter the ever-evolving landscape of cyber threats. With adversaries leveraging AI-driven techniques and exploiting the vulnerabilities inherent to virtualized and cloud-based infrastructures, static security measures are no longer sufficient. Attack vectors in 5G/6G networks range from physical layer compromises to application-layer breaches, requiring an adaptable and multi-faceted security framework. To bridge this gap, an innovative security paradigm is essential, one that combines real-time threat intelligence, machine learning, and automated response mechanisms. Such a framework must dynamically detect and mitigate threats while ensuring consistent and robust protection across all layers of the network. Embracing a proactive, rather than reactive, approach to security will be pivotal in safeguarding the next generation of wireless communications

### 1.2. Contributions

In this way, this paper introduces a hybrid security framework that integrates AI-driven threat detection with SDN/NFV-based security orchestration. The key contributions of this work include the following:

1. Cross-Layer Security Approach: A novel security architecture that enhances protection across multiple network layers, mitigating inter-slice vulnerabilities and improving threat isolation.
2. AI-Driven Threat Detection: The use of advanced AI models to detect and respond to security threats in real-time, ensuring proactive network defense.
3. Dynamic Security Orchestration: Leveraging SDN and NFV to enable real-time adaptation.
4. Simulation-Based Validation: A thorough evaluation of the proposed framework using simulation tools to measure its effectiveness in mitigating attacks and enhancing network resilience.

By integrating these components, this framework provides a proactive, intelligent security solution for 5G/6G networks, addressing both current and emerging threats.

### 1.3. Paper Outline

The remainder of this paper is organized as follows: Section 2 presents the research context, which includes an overview of network slicing and its enabling technologies, followed by a discussion of security threats in network slicing, and the role of AI/ML. Section 3 reviews the related work, analyzing current approaches to securing network slicing using SDN, NFV, and AI-based techniques. Section 4 introduces the proposed framework, detailing the hybrid cross-layer security architecture. Section 5 discusses the implementation, setup, tools, and configurations used to simulate and test the framework. Section 6 presents the results and evaluation metrics. Section 7 addresses the limitations of the proposed approach and outlines future research directions. Finally, Section 8 concludes the paper and outlines potential future work.

## 2. Research Context

The integration of network slicing, Software-Defined Networking (SDN), Network Functions Virtualization (NFV), and Artificial Intelligence/Machine Learning (AI/ML) is reshaping modern network architectures. Network slicing enables the creation of isolated virtual networks on shared infrastructure, each customized for specific services [6]. SDN provides programmable control to dynamically manage slices [7], while NFV decouples network functions from hardware, deploying them as scalable software instances [8]. Together, SDN and NFV support slicing’s flexibility for diverse use cases, from low-latency communications to IoT. However, managing these systems introduces complexity, driving the adoption of AI/ML for intelligent automation, predictive resource allocation, and security optimization [9]. For instance, AI/ML can predict traffic, detect anomalies, and automate virtual function deployment [5]. As networks evolve toward greater softwarization and intelligence, the synergy between network slicing, SDN, NFV, and AI/ML will be critical in realizing the full potential of next-generation networks.

### 2.1. Network Slicing: Fundamentals and Enabling Technologies

**Figure 1:**
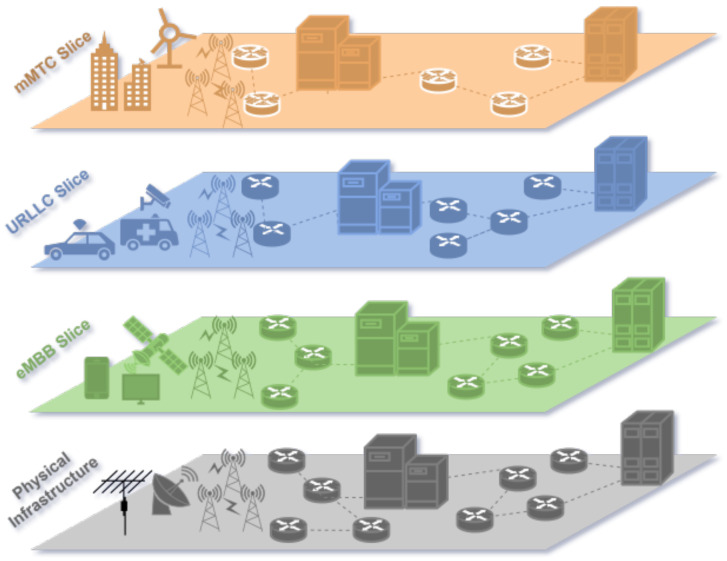
5G Network Slices: mMTC, URLLC, and eMBB on Shared Physical Infrastructure.

Network slicing employs virtualization to partition and manage computing and communication resources within a shared physical infrastructure, enabling flexible support for diverse use cases. It divides a single physical network into multiple isolated virtual networks (slices), each optimized for specific service requirements. Each slice operates independently with dedicated traffic flow, architecture, and resource provisioning, allowing providers to deliver customized services while optimizing resource utilization [10]. In 5G networks, slicing primarily supports three key service types (Figure 1):

- Enhanced mobile broadband (eMBB) focuses on providing significantly higher data rates, supporting applications like ultra-high-definition video streaming, virtual reality, and augmented reality. eMBB is designed to handle high traffic densities efficiently, catering to scenarios such as crowded urban areas and large-scale events.
- Ultra-reliable, low-latency communication (uRLLC) emphasizes achieving extremely low latency and exceptional reliability, making it ideal for mission-critical applications. It ensures minimal delay and error rates, enabling smooth and dependable communication in demanding environments.
- Massive machine-type communication (mMTC) is aimed at connecting a vast number of devices within the Internet of Things (IoT). It prioritizes power efficiency and scalability, allowing long-term operations even for battery-powered devices [11].

To fully realize the potential of network slicing, two key enabling technologies—Software-Defined Networking (SDN) and Network Functions Virtualization (NFV)—are leveraged. These technologies provide the necessary tools to efficiently manage, control, and automate network slices, ensuring dynamic provisioning and enhanced service delivery.

#### 2.1.1. Software-Defined Networking (SDN)

**Figure 2:**
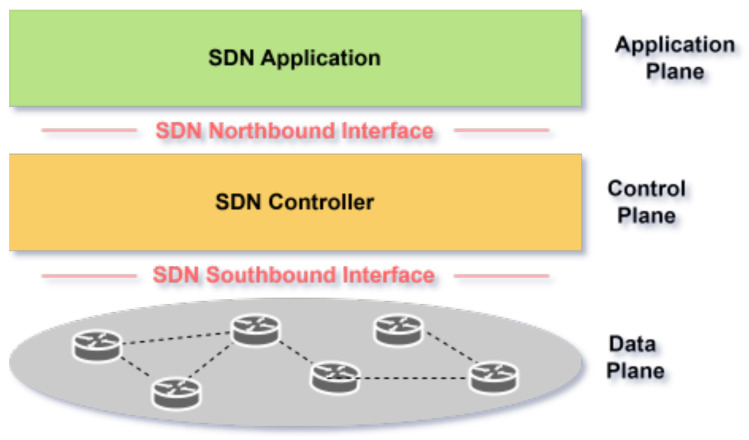
Overview of SDN Architecture.

SDN is a network architecture that decouples the control plane (decision-making) from the data plane (traffic forwarding), enabling centralized, programmable network management via a software-based controller [12,13]. This separation enhances flexibility, allowing dynamic resource allocation and efficient orchestration of network slices tailored to application requirements [12]. As illustrated in Figure 2, SDN’s architecture comprises three layers:

1. Infrastructure Layer (Data Plane): Physical/virtual devices (e.g., switches) that forward traffic based on controller instructions.
2. Control Layer: The SDN controller, which manages traffic flow, processes application-layer commands, and enforces policies.
3. Application Layer: Hosts services (e.g., VoIP, 5G slicing) that define network behavior via northbound APIs.

Communication between layers occurs through southbound interfaces (e.g., OpenFlow), which facilitate controller-to-data plane interaction, and northbound APIs, enabling application-to-controller communication for service customization [13,14]. SDN’s programmability supports dynamic scaling, isolated slices with dedicated resources, and real-time traffic adaptation. Its centralized control simplifies resource orchestration, fault detection, and performance optimization, making it pivotal for 5G and network slicing [12].

#### 2.1.2. Network Function Virtualization (NFV)

Network Functions Virtualization (NFV) revolutionizes telecommunications by decoupling network functions from proprietary hardware and implementing them as software on general-purpose servers, [15]. Working in tandem with SDN, NFV enables the flexible deployment of network functions on standardized hardware, eliminating the need for dedicated physical equipment.

**Figure 3:**
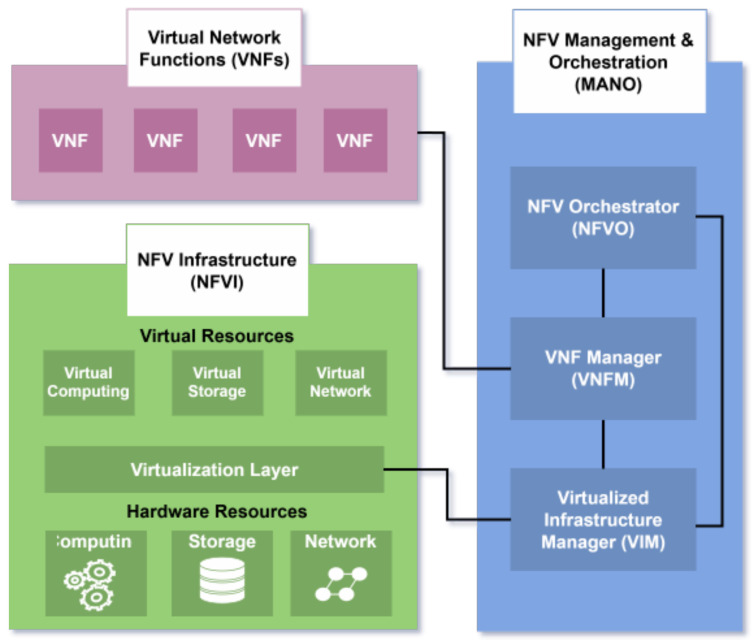
NFV Architecture.

The NFV architecture shown in Figure 3 consists of three primary components:

1. NFV Infrastructure (NFVI), which includes hardware resources (computing, storage, networking) and a virtualization layer (hypervisor) that emulates hardware and ensures VM isolation;
2. Virtual Network Functions (VNFs), software implementations of network functions running on VMs;
3. NFV Management and Orchestration (MANO), which automates resource management and lifecycle orchestration. It includes a Virtual Infrastructure Manager (VIM) controlling NFVI resources and monitoring performance; a VNF Manager handling VNF lifecycle operations and an NFV Orchestrator (NFVO) coordinating resource allocation across VIMs.

VNFs interconnect via VNF Forwarding Graphs (VNFFGs) to direct traffic flow, while Service Function Chaining (SFC) enables sequential processing through virtualized or physical functions (e.g., firewalls, load balancers). Service templates further streamline deployment by defining resource requirements for network services [16].

### 2.2. Security Threats and Challenges in 5G/6G Network Slicing

**Figure 4:**
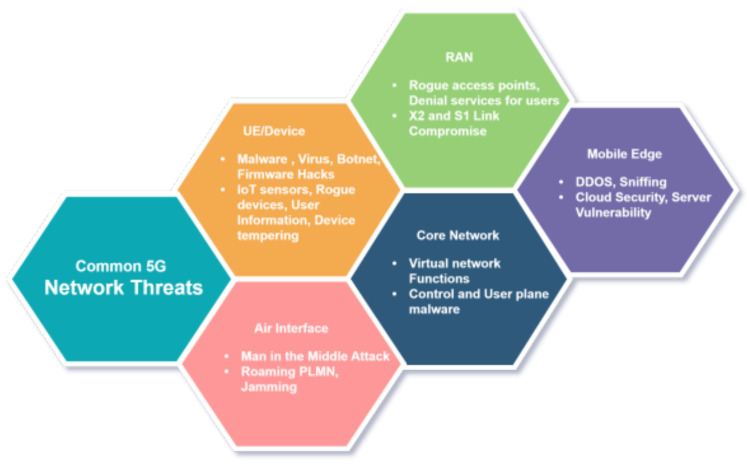
Common 5G Networks Threats across Different Architectural Components.

Network slicing introduces significant security challenges stemming from its dynamic architecture and RAN virtualization. The technology’s inherent flexibility increases vulnerability to threats, including denial-of-service attacks, traffic interception, and impersonation attacks that compromise both availability and data integrity [11,17]. These risks are magnified in multi-vendor environments where heterogeneous systems with inconsistent security implementations create exploitable gaps, potentially allowing unauthorized slice access. Poor slice isolation may enable cross-contamination between services, while shared infrastructure exposes all slices to common vulnerabilities like hypervisor exploits and rogue access points [18]. As shown in Figure 4, 5G networks face diverse threats across all components, including User Equipment (UE), RAN, Mobile Edge, and Core Network. Common attack vectors range from malware propagation and rogue access points to control-plane attacks and hypervisor exploits, each posing unique risks to slice integrity. Effective mitigation requires a secure-by-design approach integrating intrusion detection, strict access controls, and real-time monitoring within the slicing architecture itself [19,20]. The complexity escalates when managing varied security requirements across slices and their underlying SDN/NFV infrastructure, particularly for RAN-core network interfaces and multi-tenant resource sharing. Centralized management introduces additional risks, including template tampering and policy manipulation, demanding rigorous lifecycle security encompassing confidentiality, integrity, and availability principles [19,20].

### 2.3. AI/ML in Network Slicing

The growing complexity of telecommunications networks necessitates advanced management solutions, where AI and ML have emerged as critical tools for automating operations, such as network design, deployment, monitoring, and security optimization [9,21]. These technologies enable intelligent resource allocation, predictive fault detection, and dynamic configuration adjustments, addressing the diverse requirements of modern network slices. AI/ML is particularly vital for security, offering adaptive threat detection and response capabilities in the face of evolving cyber threats [9,11,22]. By analyzing historical and real-time data, ML algorithms can identify anomalies, predict attacks, and autonomously implement countermeasures, enhancing resilience against both known and zero-day threats [9,19,22]. This proactive approach is essential for 5G networks, where AI-driven predictive analytics and automated incident response safeguard slice integrity while maintaining service quality [21].

### 2.4. ML-Driven Strategies for Network Slicing

Machine learning enhances network slicing security through data-driven analysis of traffic patterns and adaptive threat mitigation. Two primary ML paradigms are employed as follows:

Supervised Learning trains models on labeled datasets to classify threats (e.g., malware, intrusions) with high accuracy, making it effective for intrusion detection systems (IDS) and spam filtering [23]. However, its reliance on labeled data and susceptibility to overfitting limit its generalization to novel threats [24].

Unsupervised Learning autonomously identifies patterns in unlabeled data, excelling in anomaly detection and uncovering emerging attack vectors [23,24]. While adaptable to unknown threats, it faces challenges in interpretability and computational complexity [25].

Together, these methods enable comprehensive security coverage—supervised learning for known threats and unsupervised learning for zero-day attacks—ensuring robust protection for dynamic network slices [21].

## 3. Related Work

The security of 5G and future networks has been an active area of research. Among the focal points, network slicing security has garnered substantial attention in recent years. Numerous studies have explored innovative approaches to fortify SDN/NFV-enabled architectures, employing advanced techniques to address emerging threats. This section provides a comprehensive review of significant contributions in this field, with a particular emphasis on the integration of AI and ML.

One significant contribution in the domain is presented in [5], where Cunha et al. explore the integration of AI, particularly Machine Learning (ML), with Software-Defined Networking (SDN) and Network Functions Virtualization (NFV) to develop advanced security mechanisms for network slicing. The study analyzes AI’s role in predictive threat detection and automated response, while examining how SDN and NFV facilitate dynamic, flexible security policy enforcement. By identifying research gaps and advocating for a holistic security framework that merges ML, SDN, and NFV, the paper emphasizes improving data confidentiality, integrity, and availability and outlines future research directions for building robust and scalable security frameworks suitable for next-generation networks. Wang et al. [26] introduces a distributed online anomaly detection framework for virtualized network slicing environments. The study proposes a decentralized one-class support vector machine (OCSVM) algorithm for detecting anomalies in physical nodes (PNs) by analyzing real-time measurements from virtual nodes. To enable distributed operation, the OCSVM problem is reformulated into decentralized quadratic programming problems using consensus constraints and solved through the alternating direction method of multipliers (ADMM). Additionally, a distributed online anomaly detection method for physical links (PLs) based on canonical correlation analysis is presented, leveraging measurement correlations between neighboring virtual nodes. The simulation results on both synthetic and real-world network datasets demonstrate the robustness and effectiveness of the proposed algorithms. The work in [27] introduces a comprehensive Security Reference Architecture for NFV. This framework provides a layered approach to securing virtualized network functions, integrating security automation with AI-powered monitoring mechanisms. Their proposed architecture ensures compliance with NFV security best practices while addressing emerging challenges such as cross-layer vulnerabilities and resource sharing risks.

Hermosilla et al. [8] advanced the discussion on securing network applications in 5G slicing by introducing an ML-driven security lifecycle approach. Their model integrates real-time threat detection, anomaly classification, and automated response mechanisms to enhance the security posture of dynamic network slices. By employing an adaptive learning mechanism, the framework ensures continuous security monitoring and proactive threat mitigation. A deep learning-based security framework was presented in [1], where the authors developed an SDN-integrated anomaly detection model for safeguarding 5G network slices. Their proposed model employs convolutional neural networks (CNN) and long short-term memory (LSTM) networks to analyze traffic patterns and detect malicious activities. The experimental results demonstrate improved QoS while maintaining high detection accuracy against adversarial attacks targeting virtualized network functions. Similarly, the Secure5G framework presented during the 2020 10th Annual Computing and Communication Workshop and Conference (CCWC) [18] leverages a deep learning-driven approach to enhance end-to-end network slicing security. Their study focuses on mitigating slice-specific security threats using a combination of deep reinforcement learning and adaptive policy enforcement mechanisms. By implementing proactive security strategies, their framework effectively reduces attack surfaces and ensures resilient slice isolation. The authors in [28] proposed T-S3RA, a traffic-aware scheduling model for secure network slicing in SDN/NFV-enabled 5G networks. The model optimizes resource allocation while considering security constraints, ensuring efficient and secure slice provisioning. Through reinforcement learning-based optimization, T-S3RA enhances resilience against slice-based attacks while maintaining optimal network performance. Further advancements in deep learning-based slicing security were presented in [29], where a hybrid deep learning framework was designed to improve the reliability of wireless network slicing in 5G environments. The study integrates multiple deep learning models, including CNN and gated recurrent units (GRU), to detect and classify security anomalies in network traffic. The proposed hybrid model enhances accuracy and robustness in real-time threat detection scenarios.

A security-aware network function sharing model for 5G slicing was introduced in [30]. The authors developed a trust-based mechanism for securely sharing virtualized network functions (VNFs) across slices while minimizing security risks. By incorporating blockchain-based authentication and AI-driven anomaly detection, their model ensures integrity and confidentiality in multi-tenant network environments. Lastly, the work in [7] presents a multi-layered defense strategy against DDoS attacks in SDN/NFV-based 5G networks. The proposed strategy integrates AI-based detection mechanisms with dynamic resource allocation to prevent service degradation due to large-scale attacks. Their framework achieves high attack detection rates while minimizing false positives, ensuring secure and efficient network operation.

## 4. Proposed Framework

Recent research has made significant strides in securing 5G network infrastructures using AI-enhanced solutions. However, as analyzed in Section 3, current frameworks often remain limited either in their architectural scope or in their adaptability to diverse, evolving threats. While existing works, such as in refs. [5,7,8], employ AI or cross-layer methods, they focus primarily on specific threat types, static configurations, or a subset of network layers.

To address these limitations, we propose a Hybrid Security Framework with Cross-Layer Integration that offers a holistic, real-time defense mechanism tailored for AI-driven 5G/6G network slicing environments. Unlike prior works, the proposed framework integrates AI/ML-based threat detection, prediction, and mitigation across all three planes—data, control, and management—while maintaining tight coordination with SDN controllers, NFV-based virtual functions, and dynamic slice orchestration.

Our architecture introduces several key innovations that distinguish it from existing approaches. First, it offers full cross-layer integration, with security mechanisms operating across the data, control, and management planes. This allows the system to detect traffic-level anomalies, control-plane manipulation, and orchestration-level threats in a unified manner. Second, the framework is designed for real-time adaptability, enabling continuous threat monitoring and automatic mitigation actions, such as VNF reallocation, slice isolation, and SDN flow rule adjustments without human intervention. Furthermore, it incorporates explicit slice-awareness, allowing security policies to be tailored per slice and dynamically adjusted based on the slice’s context, function, and threat exposure. Finally, through context-aware orchestration, the system maintains a feedback loop between AI-driven threat intelligence and slice/network management decisions, empowering the network to proactively adjust its defense posture in response to evolving attack patterns.

A comparative analysis with closely related works is shown in Table 1. As highlighted, while other solutions provide partial cross-layer support or target specific security aspects, our framework uniquely delivers a unified, intelligent, and self-adaptive security model for sliced, virtualized, and software.

**Figure 5:**
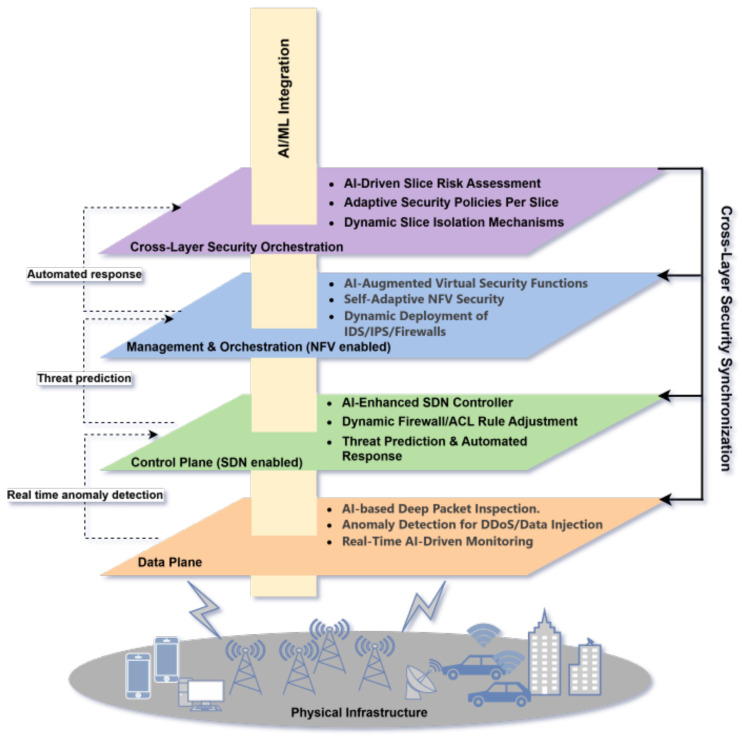
Proposed Framework Architecture.

The architecture, as illustrated in Figure 5 integrates security across the Data Plane, Control Plane, Management and Orchestration Security, and Cross-Layer Security Orchestration layers, ensuring a holistic defense strategy. In the Data Plane, real-time threat monitoring and anomaly detection mechanisms are employed, utilizing AI-driven deep packet inspection to counter malicious traffic and DDoS attacks. The Control Plane, powered by SDN, facilitates proactive threat prediction and adaptive routing via AI-augmented controllers, alongside dynamic firewall adjustments. Meanwhile, the Management and Orchestration Security layer utilizes NFV to deploy virtual security functions, such as adaptive intrusion detection systems and firewalls, tailored to the needs of each network slice. The Cross-Layer Security Orchestration ensures coherence by dynamically adapting security policies across slices, leveraging AI for risk assessment and isolation of compromised slices. This integration of AI/ML across all layers enables continuous learning and adaptation, empowering the framework to detect, predict, and respond to threats in real time. By ensuring interconnected security mechanisms, the architecture achieves both robust protection and optimal network performance, offering an intelligent and adaptive solution to the security challenges posed by modern network slicing environments.

## 5. Implementation and Evaluation

The framework is implemented using a combination of AI/ML tools, SDN platforms, and NFV technologies, simulating a resilient and intelligent security environment for network slicing.

### 5.1. Dataset and Preprocessing

The UNSW-NB15 dataset is utilized, which is a modern network intrusion detection benchmark developed at UNSW Canberra. The dataset consists of approximately 2.54 million network flow records, each annotated with 49 features and labeled as either normal or one of nine attack types: Fuzzers, Analysis, Backdoors, DoS, Exploits, Generic, Reconnaissance, Shellcode, and Worms. The dataset is divided into two official splits: 175,341 records for training and 82,332 for testing. Our ML-based detection task is framed in two ways:

1. **Binary classification:** Distinguishing between normal and attack traffic (for model comparison).
2. **Multiclass classification:** Classifying traffic into one of the nine attack types (for final deployment).

The primary objective is to accurately detect anomalies while minimizing false positives, in order to support real-time mitigation.

Before modeling, a consistent preprocessing pipeline is applied to handle mixed data types. Irrelevant fields are removed (such as record IDs and redundant binary labels), and the focus is on the multiclass attack_cat label. Using scikit-learn’s ColumnTransformer, features are separated into numeric and categorical sets. Numerical features (e.g., byte counts, rates, etc.) were scaled or used directly, while categorical features (such as protocol or service types) were converted via one-hot encoding. One-hot encoding creates a binary indicator column for each category, ensuring the model does not assume any ordering among categories. (For example, the protocol field values “TCP”, “UDP” become separate 0/1 columns). The target column attack_cat was label-encoded into integer class labels for modeling, while preserving the mapping to original names for interpretation. In practice, we used a scikit-learn Pipeline that first applied a OneHotEncoder to categoricals and optionally a scaler to numericals (within a ColumnTransformer), then fed the output into the classifier. This ensures that future data can be transformed in the same way. All preprocessing objects (encoder, scaler) were saved for reuse in deployment.

Once preprocessing was finalized, the transformed dataset was used to train a suite of machine learning classifiers. The models explored include Decision Tree, Random Forest, K-Nearest Neighbors (KNN), and XGBoost, selected for their performance in classification tasks and compatibility with streaming or near-real-time inference environments. For binary classification, models were trained to distinguish between normal and malicious traffic; in the multiclass setting, models aimed to classify traffic into one of the nine predefined attack categories.

All models were trained using the official training split of the UNSW-NB15 dataset, with stratified sampling to preserve class distributions. Hyperparameters were tuned through manual experimentation to balance complexity and inference speed. The XGBoost model, configured with the multi:softmax objective for multiclass classification, was ultimately chosen for integration into the security framework due to its balance of prediction accuracy, training efficiency, and scalability.

To support deployment, both the preprocessing pipeline and the final trained model were serialized using joblib. This enables seamless integration into the cross-layer framework, allowing real-time packet statistics to be transformed and classified consistently within the SDN-driven control loop. The model’s inference output can then trigger appropriate mitigation policies at the network level.

### 5.2. SDN-Based Slice Monitoring and Response

Within the proposed framework, the Software-Defined Networking (SDN) layer plays a central role in enabling dynamic threat response and fine-grained network control. By decoupling the control and data planes, SDN provides a programmable environment where flow-level decisions can be made intelligently based on real-time traffic insights. Once the trained machine learning model is embedded within the SDN controller (such as the Ryu controller), it continuously analyzes incoming flow statistics to detect abnormal patterns indicative of potential attacks. Upon identifying anomalous behavior, the controller initiates immediate mitigation actions through the OpenFlow protocol. These actions include the following:

- Traffic rerouting: Redirecting suspicious flows to alternative paths or to security inspection points to prevent potential service disruption.
- Rate limiting: Throttling the bandwidth allocated to malicious or suspicious flows to contain the impact.
- Slice isolation: Temporarily isolating the affected slice from others to prevent lateral movement of threats and preserve overall network integrity.

This real-time mitigation loop ensures that attacks are addressed promptly and locally, reducing the need for manual intervention while maintaining the performance and security of unaffected network slices. The programmability and centralized visibility offered by SDN make it an essential enabler of adaptive and automated threat response in the proposed cross-layer security architecture.

### 5.3. NFV Integration and VNF Deployment

The implementation of the Network Function Virtualization (NFV) layer involved the development and deployment of a comprehensive system designed to manage virtualized network functions (VNFs) within a simulated network environment. The process began with the creation of a management module to orchestrate the deployment of VNFs, leveraging containerization technology to ensure flexibility and scalability. This module was enhanced with a monitoring framework to track the performance and status of the VNFs, enabling the real-time assessment of system health and resource utilization. An auxiliary inference component was integrated to support threat detection, providing analytical capabilities to identify potential network anomalies and the system’s responsiveness to evolving threat conditions. Furthermore, the orchestration of VNFs is closely aligned with the AI-driven detection and SDN-based response mechanisms, ensuring end-to-end coordination across the entire security stack.

### 5.4. Cross-Layer Security Orchestration

The cross-layer security orchestration module serves as the coordination core of the proposed framework, enabling intelligent decision-making and synchronized responses across the data, control, and management layers. Its implementation is designed to ensure that information flows bidirectionally between these layers in real time, allowing for adaptive and context-aware mitigation strategies.

This cross-layer logic is implemented as a lightweight service running alongside the SDN controller and NFV orchestrator, ensuring minimal latency in control signaling. The orchestration engine uses predefined security policies, dynamically updated risk scores, and AI-derived anomaly patterns to guide its decisions. Communication between layers is secured via TLS, and control actions are logged and auditable to ensure traceability and compliance with network security standards.

When a threat is detected at any layer, the orchestrator performs correlation analysis to determine its potential impact across other layers. For example, an anomaly in flow behavior may be cross-verified with rising CPU usage on a VNF or degradation in slice latency. If the threat is confirmed, a coordinated response is generated: the SDN controller may be instructed to reroute or block specific flows, the NFV layer may be prompted to scale up VNFs or instantiate new ones, and security policies may be updated at the orchestration level to isolate the affected slice or adjust firewall rules.

By enabling real-time synchronization across all planes, the cross-layer orchestrator ensures that responses are both holistic and optimized, effectively enhancing the resilience and adaptability of the entire network slicing environment.

## 6. Results

To validate the effectiveness of the proposed AI-driven cross-layer security framework for 5G/6G networks, an initial evaluation was conducted using the UNSW-NB15 dataset. Multiple machine learning models—including Random Forest, SVM, XGBoost, Extra Trees, and Gradient Boosting—were trained and tested to assess their ability to detect a wide range of cyberattacks. This experimental setup provides a representative assessment of the framework’s detection performance and responsiveness in real-time, under practical conditions.

It is important to note that the results presented here reflect the current stage of implementation and are based on a controlled configuration that emphasizes core framework functionalities. Ongoing work involves extending the evaluation to larger and more diverse datasets and completing the full SDN/NFV integration for dynamic security service deployment. These extended evaluations will be included in a future version of this study.

### 6.1. Model Comparison

**Figure 6:**
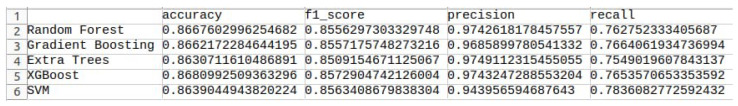
Comparison of Classification Performance Metrics.

We evaluated several classifiers on the binary detection task. Specifically, we trained Decision Tree, Random Forest, K-Nearest Neighbors (KNN), and XGBoost models (also testing Extra Trees and Gradient Boosting for completeness). We measured standard metrics: accuracy, precision, recall, and F1-score on the test set. Figure 6 summarizes the core results for key models:

The ensemble models (Random Forest, XGBoost) achieved the highest accuracy (86–87%) and balanced precision/recall as shown in the figure. In contrast, simpler models like Decision Trees and KNN yielded lower accuracy (71–74%) on this dataset. This trend is consistent with published results: for example, Khan et al. reported 74.87% accuracy for a random forest and 74.22% for a decision tree on UNSW-NB15. In our results, XGBoost slightly outperformed Random Forest on F1-score, aligning with the literature’s finding that XGBoost often has the best overall balance of metrics.

**Figure 7:**
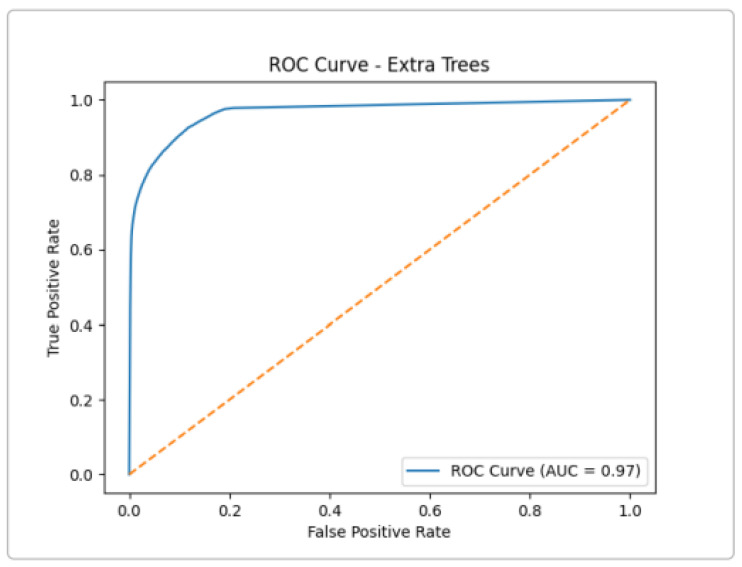
ROC Curve for The Extra Trees Classifier (binary detection).

The Extra Trees model attained an AUC of about 0.97 (Figure 7), indicating strong discriminative ability between normal and attack traffic. The curve’s steep rise (TPR) at a low FPR shows that most attacks are correctly identified with few false alarms. High AUC (near 1.0) reflects that the model makes very few mistakes across threshold choices. These results match the high precision and recall reported above for ensemble methods.

**Figure 8:**
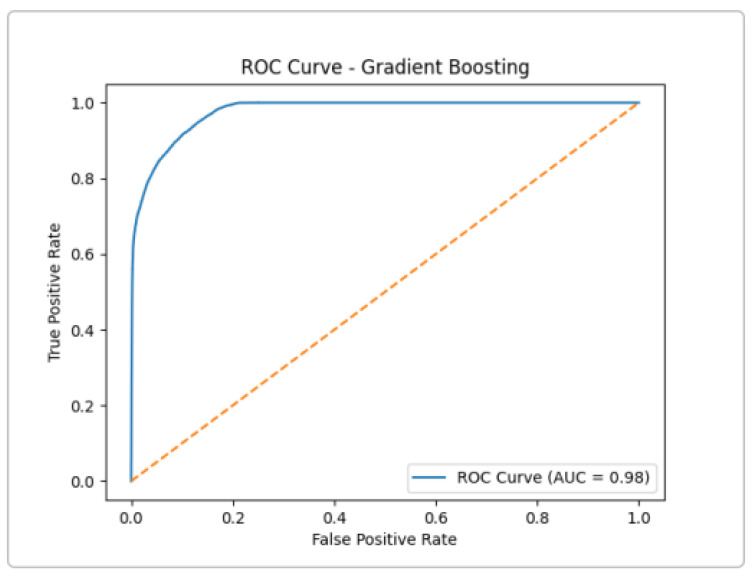
ROC Curve for The Gradient Boosting Classifier.

Gradient Boosting achieved AUC ≈0.98 (Figure 8), the highest of the ensemble group. This near-perfect ROC suggests that Gradient Boosting can capture complex patterns in the data, at the cost of longer training time. In practice, its performance was very close to XGBoost.

**Figure 9:**
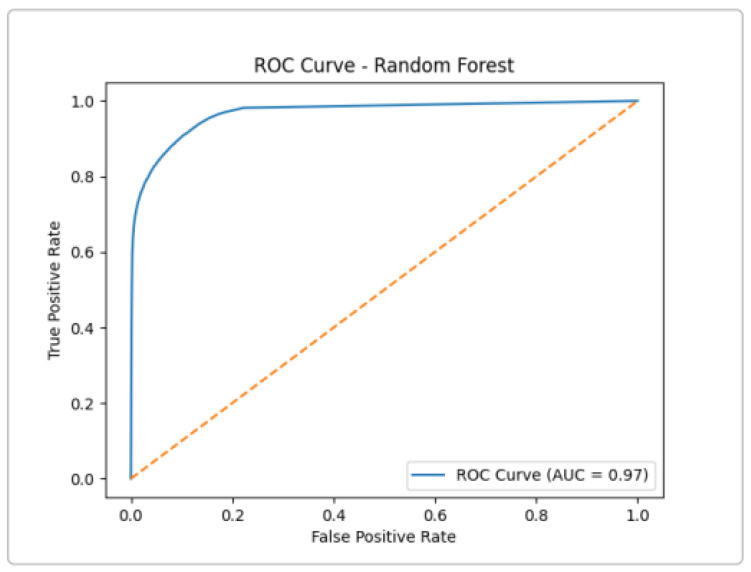
ROC curve for the Random Forest classifier.

Like Extra Trees, Random Forest shows excellent separation, with a very low false positive rate even at a high true positive rate (Figure 9). In summary, all three tree-based models achieved an AUC ≈ 0.97–0.98 on UNSW-NB15, confirming that ensemble methods are highly effective for attack detection.

#### 6.1.1. Final Model Development

For the final deployed model, we focused on multi-class classification of attack categories using XGBoost. We instantiated an XGBClassifier with objective = ‘multi:softmax’ to directly predict the attack category. Hyperparameters were tuned (via cross-validation and grid search) to find a good setting: for example, we used about 300 trees (n_estimators = 300), a max_depth = 8, and a moderate learning_rate = 0.05. We trained on the 175 K examples (with a stratified train/test split to preserve class ratios) and evaluated on the 82 K test set. All preprocessing artifacts (the fitted one-hot encoder, any scalers, etc.) were saved to disk (e.g., as preprocessing_artifacts.pkl) so that incoming traffic can be transformed the same way at inference. The trained XGBoost model was serialized (xgboost_multiclass_model.pkl) for later deployment. After training, we examined the feature importances provided by XGBoost. The most important features were typically flow-level statistics (such as source/destination byte counts, packet rates, etc.), which is consistent with UNSW-NB15’s focus on flow features.

#### 6.1.2. Confusion Matrix Analysis

The confusion matrix provides a detailed breakdown of the model’s performance at the class level. Key observations from the confusion matrix are as follows:

- **True Positives (TP)**: The classifier correctly identified instances of normal traffic and instances of attack traffic. These true positives confirm that the model effectively distinguishes between the two classes.
- **False Positives (FP)**: The number of false positives was extremely low, indicating that the classifier rarely misclassified normal traffic as attack traffic. This result contributes to the high precision and overall reliability of the model.
- **False Negatives (FN)**: Similarly, the model showed minimal false negatives, meaning very few attack instances were misclassified as normal traffic. This is crucial for maintaining high recall and ensuring the model does not overlook security threats.
- **True Negatives (TN)**: The model also demonstrated a strong ability to correctly classify normal traffic, further reinforcing its effectiveness and reliability.

#### 6.1.3. Overall Model Performance

To evaluate the robustness and generalization capability of the proposed framework, we conducted a comprehensive comparison of five machine learning classifiers: Random Forest, Support Vector Machine (SVM), XGBoost, Extra Trees, and Gradient Boosting. Each model was trained and tested on the multiclass version of the UNSW-NB15 dataset, which includes normal traffic and nine distinct attack categories. This setup allows for a more realistic assessment of the framework’s ability to accurately distinguish between a diverse range of threats in a real-world network environment. The models were evaluated using confusion matrices, which provide detailed insights into classification accuracy for each attack type, as well as the distribution of misclassifications. This analysis highlights the strengths and weaknesses of each model in handling imbalanced data, detecting rare attack types, and minimizing both false positives and false negatives across multiple threat categories.

**Figure 10:**
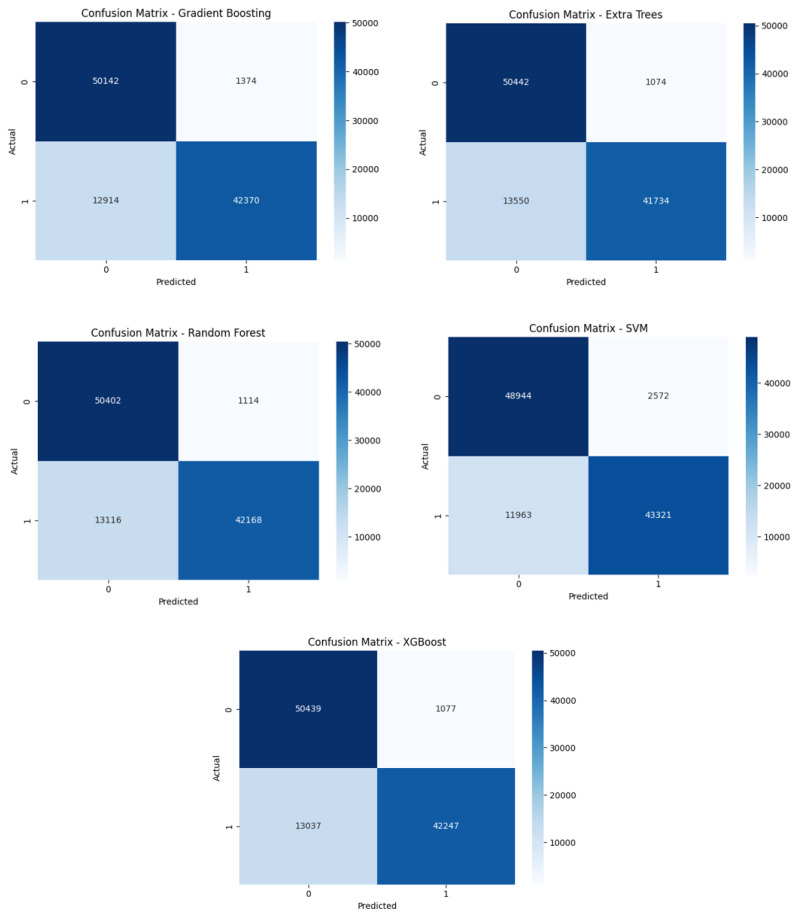
Confusion Matrices for Classification Results for All Models.

From the confusion matrices observed in Figure 10, the main differences were in the trade-off between false positives and false negatives. For example, XGBoost had a slightly higher true positive rate (catching more attacks) at the cost of a few more false alarms, whereas Random Forest was very conservative (few false positives). In general, we note that ensemble methods like Random Forest and XGBoost topped performance: XGBoost achieved the highest overall accuracy and F1-score. Decision Tree and KNN lagged behind due to overfitting (DT) or sensitivity to high-dimensional data (KNN).

### 6.2. Evaluation Results

The final XGBoost model achieved a 75% accuracy on the multi-class test set. The table below is an example classification report (precision, recall, F1) for each class:

As expected, Table 2 shows that the majority of classes like Normal, Exploits, and Reconnaissance had the highest precision/recall (mostly 75–90%), whereas rare categories (Worms, Shellcode, etc.) showed lower recall (30–50%). The weighted-average F1-score was in the order of 75–80%. This performance reflects both the model’s ability and the data’s imbalance. The (binary) confusion matrix for normal vs. attack was strongly diagonal: most normal traffic was correctly labeled (true negatives) and most attacks (true positives) were caught. Misclassifications (false negatives) mostly occurred in minority attacks that the model saw few examples of. In line with this, we saw that rare attack classes had the most errors. For instance, many Worm or Shellcode flows were misclassified as other attacks, explaining their lower recall. Overall, false positives were low for normal traffic, meaning few benign flows were flagged as attacks. This balance led to high precision for the ‘attack’ side.

## 7. Limitations and Future Directions

While the proposed AI-driven cross-layer security framework demonstrates promising detection capabilities and architectural flexibility, several limitations must be acknowledged.

First, the integration of AI models into real-time network environments introduces computational overhead, particularly during feature extraction, model inference, and dynamic policy enforcement. Although tree-based models such as Random Forest and XGBoost offer relatively efficient prediction times, their performance may still degrade under high-throughput traffic conditions, especially when scaling to large network slices or multi-tenant infrastructures.

Second, latency implications arise from the interplay between the detection layer, SDN-based control decisions, and NFV-based mitigation orchestration. The multi-stage pipeline—consisting of packet inspection, classification, policy generation, and VNF deployment—can accumulate delays that impact time-sensitive applications or ultra-reliable low-latency communication (URLLC) scenarios in 5G/6G.

Furthermore, the current implementation relies on simulated or small-scale testbeds, which limits the generalizability of results. Scalability and performance under real-world, large-scale traffic loads remain to be fully validated.

Future work will address these limitations through optimized model compression techniques, latency-aware orchestration strategies, and large-scale real-world testing to ensure the framework’s practicality in production-grade 5G/6G deployments.

## 8. Conclusions

This work presented a cross-layer, AI-driven security framework designed to enhance the protection of network slicing environments in 5G and next-generation networks. By integrating machine learning-based anomaly detection with SDN-enabled real-time mitigation and NFV-based orchestration, the proposed system addresses the increasing demand for intelligent, adaptive, and slice-aware security solutions in modern programmable infrastructures.

The framework operates across three synchronized layers: a monitoring layer for flow-level traffic collection, an intelligence layer powered by traditional and deep learning models for anomaly detection, and a control/orchestration layer that utilizes SDN and NFV to enforce dynamic mitigation and resource allocation policies. The current implementation includes the AI-based detection layer and its integration with the SDN controller for real-time threat response. The full NFV integration and cross-layer synchronization are ongoing and will enable dynamic VNF deployment and orchestration in future phases.

Evaluation was conducted using the UNSW-NB15 dataset. The model was tested against nine distinct attack categories, with a classification report showing its ability to detect diverse threat types. ROC curves, AUC scores, and multiple-run analysis validated the statistical robustness of the results.

This research contributes a scalable and intelligent security architecture that unifies AI, SDN, and NFV technologies. It lays a strong foundation for resilient, autonomous, and context-aware defense mechanisms tailored for the dynamic and complex demands of 5G/6G network slicing environments.

## Figures and Tables

**Table 1:** Comparative Analysis of Related Cross-Layer Security Frameworks.

Feature/Paper	[5]	[7]	[8]	Our Framework
AI/ML Integration	Yes (General detection)	Yes (Focused on DDoS)	Yes (App lifecycle)	Multi-plane prediction & adaptation
Cross-Layer Design	Ctrl + Data	Ctrl + Data	Partial (App-level focus with limited control integration)	Full (Data, Control, Mgmt)
Scope of Threats	General intrusions	DDoS only	Lifecycle attacks	Broad, evolving threats
Real-Time Response	Periodic	Predefined rules	Limited	Continuous & adaptive
Slice Awareness	Mentioned	Indirect	No	Explicit & dynamic slicing
VNF Management	Partial NFV use	Static config	No	Dynamic, AI-informed VNF control
Novelty	AI in SDN/NFV	Multi-layer DDoS defense	Lifecycle-focused ML	Holistic, slice-driven AI defense

**Table 2:** Example Classification Report.

Class	Precision (%)	Recall (%)	F1-Score (%)	Support
Normal	85	90	87	56,000
Exploits	80	75	77	15,000
Reconnaissance	78	80	79	20,000
Generic	82	70	76	10,000
DoS	88	82	85	12,000
Others (combined rare classes)	60	40	48	1332
**Accuracy/Avg**	75 (acc)		78 (weighted avg)	82,332

## Data Availability

The original contributions presented in this study are included in the article. Further inquiries can be directed to the corresponding authors.

## Abbreviations

The following abbreviations are used in this manuscript:

5G	Fifth Generation
6G	Sixth Generation
AI	Artificial Intelligence
AR	Augmented Reality
CNN	Convolutional Neural Networks
DDoS	Distributed Denial of Service
eMBB	Enhanced Mobile Broadband
GRU	Gated Recurrent Units
ID/IP	Intrusion Detection/Intrusion Prevention
IoT	Internet of Things
ITU	International Telecommunication Union
LD	Linear Dichroism
LSTM	Long Short-Term Memory
ML	Machine Learning
mMTC	Massive Machine-Type Communication
NFV	Network Function Virtualization
NFV MANO	Network Function Virtualization Management and Orchestration
NFVI	Network Function Virtualization Infrastructure
PLMN	Public Land Mobile Network
RBAC	Role-Based Access Control
RAN	Radio Access Network
SFC	Service Function Chaining
SDN	Software-Defined Networking
TLA	Three-Letter Acronym
THz	Terahertz
TLS/SSL	Transport Layer Security/Secure Sockets Layer
URLLC	Ultra-Reliable Low-Latency Communication
VIM	Virtual Infrastructure Manager
VNF	Virtual Network Function
VNFC	Virtual Network Function Component
VNFFG	Virtual Network Function Forwarding Graph
VoIP	Voice over Internet Protocol
WAN	Wide Area Network
